# Chromatin alterations during the epididymal maturation of mouse sperm refine the paternally inherited epigenome

**DOI:** 10.1186/s13072-021-00433-4

**Published:** 2022-01-06

**Authors:** Yudhishtar S. Bedi, Alexis N. Roach, Kara N. Thomas, Nicole A. Mehta, Michael C. Golding

**Affiliations:** Department of Veterinary Physiology & Pharmacology, College of Veterinary Medicine and Biomedical Sciences, Texas A&M University, College Station, Texas 77843-4466 USA

**Keywords:** Sperm, Chromatin structure, Epididymal maturation, Gene enhancers, H3K27ac, H3K9me2, Histone h3.3, Developmental programming, Paternal epigenetic inheritance

## Abstract

**Background:**

Paternal lifestyle choices and male exposure history have a critical influence on the health and fitness of the next generation. Accordingly, defining the processes of germline programming is essential to resolving how the epigenetic memory of paternal experiences transmits to their offspring. Established dogma holds that all facets of chromatin organization and histone posttranslational modification are complete before sperm exits the testes. However, recent clinical and animal studies suggest that patterns of DNA methylation change during epididymal maturation. In this study, we used complementary proteomic and deep-sequencing approaches to test the hypothesis that sperm posttranslational histone modifications change during epididymal transit.

**Results:**

Using proteomic analysis to contrast immature spermatozoa and mature sperm isolated from the mouse epididymis, we find progressive changes in multiple histone posttranslational modifications, including H3K4me1, H3K27ac, H3K79me2, H3K64ac, H3K122ac, H4K16ac, H3K9me2, and H4K20me3. Interestingly, some of these changes only occurred on histone variant H3.3, and most involve chromatin modifications associated with gene enhancer activity. In contrast, the bivalent chromatin modifications, H3K4me3, and H3K27me3 remained constant. Using chromatin immunoprecipitation coupled with deep sequencing, we find that changes in histone h3, lysine 27 acetylation (H3K27ac) involve sharpening broad diffuse regions into narrow peaks centered on the promoter regions of genes driving embryonic development. Significantly, many of these regions overlap with broad domains of H3K4me3 in oocytes and ATAC-seq signatures of open chromatin identified in MII oocytes and sperm. In contrast, histone h3, lysine 9 dimethylation (H3K9me2) becomes enriched within the promoters of genes driving meiosis and in the distal enhancer regions of tissue-specific genes sequestered at the nuclear lamina. Maturing sperm contain the histone deacetylase enzymes HDAC1 and HDAC3, suggesting the NuRD complex may drive some of these changes. Finally, using Western blotting, we detected changes in chromatin modifications between caput and caudal sperm isolated from rams (*Ovis aries*), inferring changes in histone modifications are a shared feature of mammalian epididymal maturation.

**Conclusions:**

These data extend our understanding of germline programming and reveal that, in addition to trafficking noncoding RNAs, changes in histone posttranslational modifications are a core feature of epididymal maturation.

**Supplementary Information:**

The online version contains supplementary material available at 10.1186/s13072-021-00433-4.

## Background

Although gestational stressors have well-established effects on offspring growth and development, preconception exposures have only recently emerged as having a critical influence on the health and fitness of the next generation. Indeed, an increasing number of studies reveal the potential of parental lifestyle choices and exposure history to impart inter- or transgenerational phenotypic changes to the offspring [1]. Therefore, understanding how and when germline programming occurs is essential to deciphering the developmental origins of disease and, concerning preconception male exposures, addressing a major blind spot in the field of developmental toxicology.

Male germ cell specification and differentiation exhibit dramatic changes in chromatin structure, ranging from erasure and re-establishment of DNA methylation during the embryonic phases to the critical role of posttranslational histone modifications during meiosis (reviewed [2]). During sperm production, stage-specific alterations in chromatin structure coincide with the trafficking of multiple histone variants, which prepare sperm for transcriptional quiescence and nuclear compaction [3]. Finally, during the late stages of spermatogenesis, where germ cells differentiate into round spermatids (spermiation), most histones are evicted and replaced with protamines [4].

Despite the essential role of protamines in nuclear compaction, a small subset of the paternal genome remains packaged in nucleosomes. Depending on the method of analysis, these nucleosome-enriched regions colocalize with regulatory regions of developmentally crucial genes [5–12] or gene-poor domains enriched in repetitive elements [13–16]. The retention of nucleosomes, along with region-specific patterns of posttranslational modifications, is hypothesized to contribute to the establishment of the embryonic transcriptional program [17–19]. In support of this assertion, markers of bivalent poised chromatin can be traced from the establishment of the male germline during early embryonic development through meiosis into fertilization-competent sperm [20].

During epididymal transit, sperms receive additional epigenetic information in the form of secreted noncoding RNAs [21]. Furthermore, substantial evidence now indicates that environmental stressors alter the noncoding RNA profile of sperm, transmitting an epigenetic memory of these experiences to the offspring [22]. Notwithstanding this recent discovery, the established dogma remains that all facets of chromatin-based epigenetic programming are complete before exiting the testis [2, 23, 24]. This dogma has persisted because the nuclear compaction established during spermiation is thought to physically exclude enzyme complexes involved in chromatin modification. However, studies examining short-term exposures in rodents [25] and clinical studies comparing sperm from men one week before and one week after bariatric surgery suggest the DNA methylation profile of sperm is labile during the final stages of maturation [26]. Indeed, studies in mice examining epididymal maturation have identified a small number of loci that exhibit changes in DNA methylation [27, 28], although others suggest patterns are primarily static [29]. These observations, along with the recent identification of differential patterns of histone H3 enrichment across epididymal transit [30], raise the prospect that histone posttranslational modifications may also change during sperm maturation. Large-scale proteomic studies have reported changes in select histone posttranslational modifications between elongating spermatids and mature sperm [31]. However, these previous studies never determined whether changes in posttranslational histone modifications occurred in the testis or during epididymal transit. This question is significant as it implies that acute, periconceptional male exposures could alter aspects of chromatin-based epigenetic programming and that epididymal noncoding RNAs may play a role in directing this process. Using a mouse model, we investigated the hypothesis that sperm chromatin posttranslational modifications change during epididymal transit.

## Results

### Comparison of sperm histone variants and posttranslational modifications between the caput and caudal portions of the mouse epididymis

To achieve an unbiased assessment of changes in chromatin structure, we separately isolated sperm from the caput (initial segment) and cauda portions of epididymides taken from ~ 12-week-old C57BL/6 N males and acid-extracted histones. Using nano-liquid chromatography followed by triple quadrupole mass spectrometry (LC/MS), we measured histone composition and abundance in pooled samples isolated from at least 10 million caput and cauda sperm. Our analyses quantified the posttranslational modifications at 30 different residues across histones H2A, H2A1 H2A3, H3.1, H3.3, and H4. We find that most of the residues examined (19/30) are unmodified in both caput and cauda sperm (Additional file 1: Table S1). Notable exceptions were histone H3 lysine 9 (H3K9),^1^ histone H3.1 and H3.3 lysine 27, and lysine 36 (H3K27 and H3K36), as well as histone H4 lysine 20 (H4K20), which were all at least 50% modified. Consistent with previous reports examining human and mouse sperm [31], we could only identify trace amounts of histone H1.4.

**Fig. 1:**
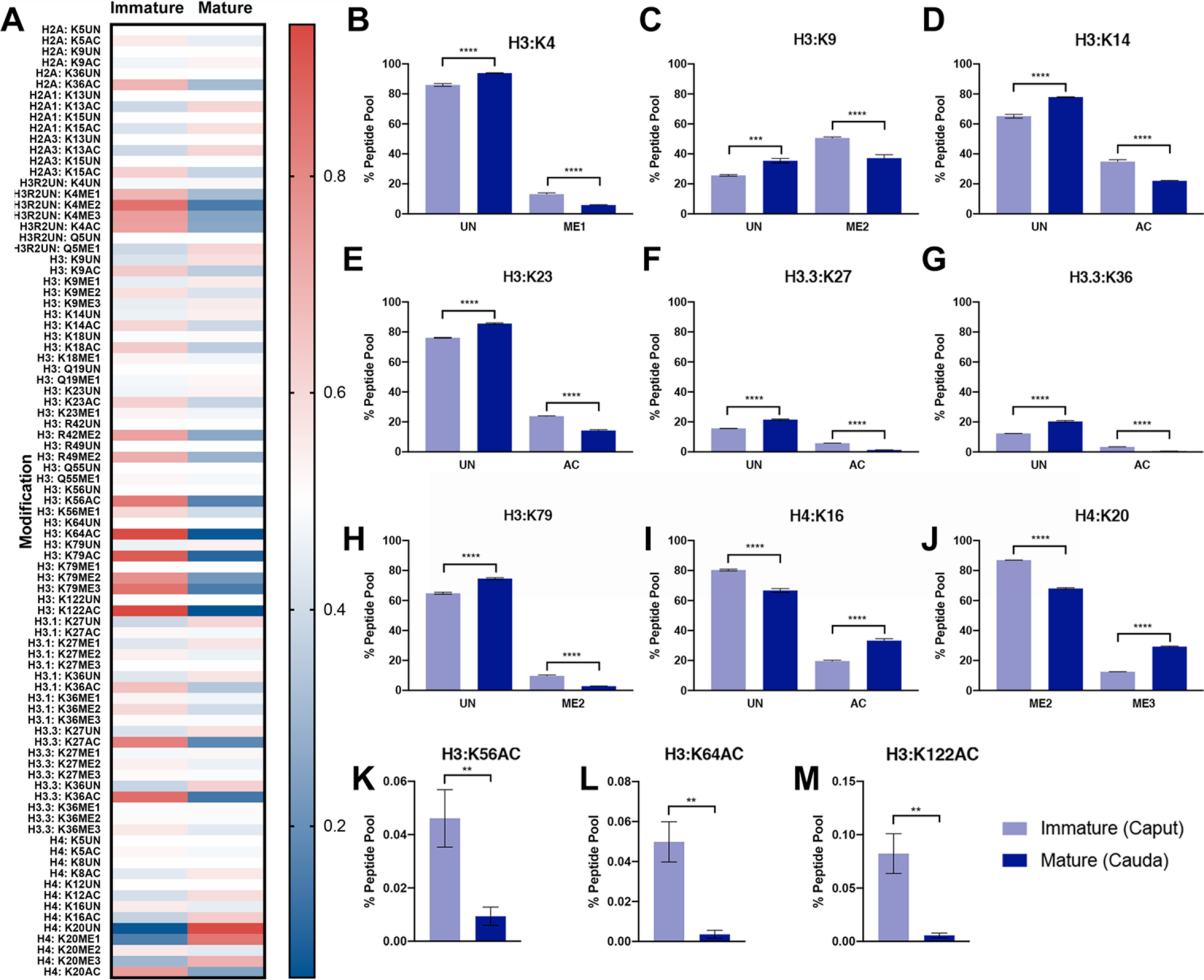
Gains and losses of select histone posttranslational modifications during epididymal maturation of mouse sperm. **A** Heatmap comparing the LC/MS-derived percentages for each peptide transition as a fraction of the total peptide pool (all modifications + unmodified peptides = 100%). Data represent the average of three independent runs obtained from a pooled sample of 10 million caput and cauda sperm. **B**–**M** Bar graphs contrasting gains and losses of unmodified (UN) histones with the levels of lysine acetylation (AC), monomethylation (ME1) dimethylation (ME2), and trimethylation (ME3) in sperm isolated from the caput and caudal portions of the epididymis. Error bars represent the SEM, *n* = 3 replicates, ** *p* < 0.01, *** *p* < 0.001, **** *p* < 0.0001

To determine if alterations in chromatin structure occur during sperm maturation, we examined changes in the relative abundance of the measured posttranslational modifications between caput and cauda sperm. These analyses identified 12 different residues that exhibited either a ~fivefold or greater difference in relative abundance or a shift of greater than ~ 10% within the peptide pool (Fig. 1A). These included changes at histone H3, lysine 4 (H3K4), lysine 9 (H3K9), lysine 14 (H3K14), lysine 23 (H3K23), lysine 27 (H3K27), lysine 36 (H3K36), lysine 56 (H3K56), lysine 64 (H3K64), lysine 79 (H3K79), and lysine 122 (H3K122) as well as histone H4 lysine 16 (H4K16), and lysine 20 (H4K20). We did not observe any changes for the isoforms of histone H2.

In the transition from caput spermatozoa to cauda sperm, decreases in most posttranslational modifications were balanced by equivalent gains in the unmodified form of the histone (Fig. 1B–M). As examples, between caput and cauda sperm, we observed an ~ 8% decrease in H3K4 monomethylation, which was offset by an 8% increase in unmodified H3K4 and a ~ 10% decline in H3K9 dimethylation equipoised by a near equivalent increase in unmodified H3K9 (Fig. 1B–C). Similarly, for histone H3 lysines 14, 23, and 27, ~ 5% to 13% decreases in acetylation were accompanied by equivalent increases in the unmodified forms of these residues within the peptide pool (Fig. 1D–F). The more substantial magnitude shifts in the acetylation of H3K27 appeared on histone H3.3 and not the H3.1/H3.2 isoforms (LC/MS analysis cannot distinguish H3.1 or H3.2). In contrast, histone H4 lysine 16 (H4K16ac) acetylation increased 13%, while unmodified H4K16 decreased by 13% (Fig. 1I). The remaining changes in histone posttranslational modifications represented shifts in distributions between multiple posttranslational modifications. As examples, H3K36 and H3K79 exhibited losses in acetylation and methylation, accompanied by an increased abundance of the unmodified peptide (Fig. 1G–H). At the same time, H4K20 displayed an 18% decline in the dimethylated form and an equivalent increase in the trimethylated form (Fig. 1J). Although less than 1% of the peptide pool, we observed 5- to 15-fold decreases in the acetylation of histone H3, lysine 56, 64, and 122 (H3K56, H3K64, and H3K122, Fig. 1K–M). Notably, trimethylation of histone H3 lysine 4 (H3K4me3) and lysine 27 (H3K27me3) were constant during the transition from the caput to caudal region of the epididymis (Fig. 1A).

### Changes in the profile of posttranslational modifications associated with enhancer function during epididymal maturation

**Fig. 2:**
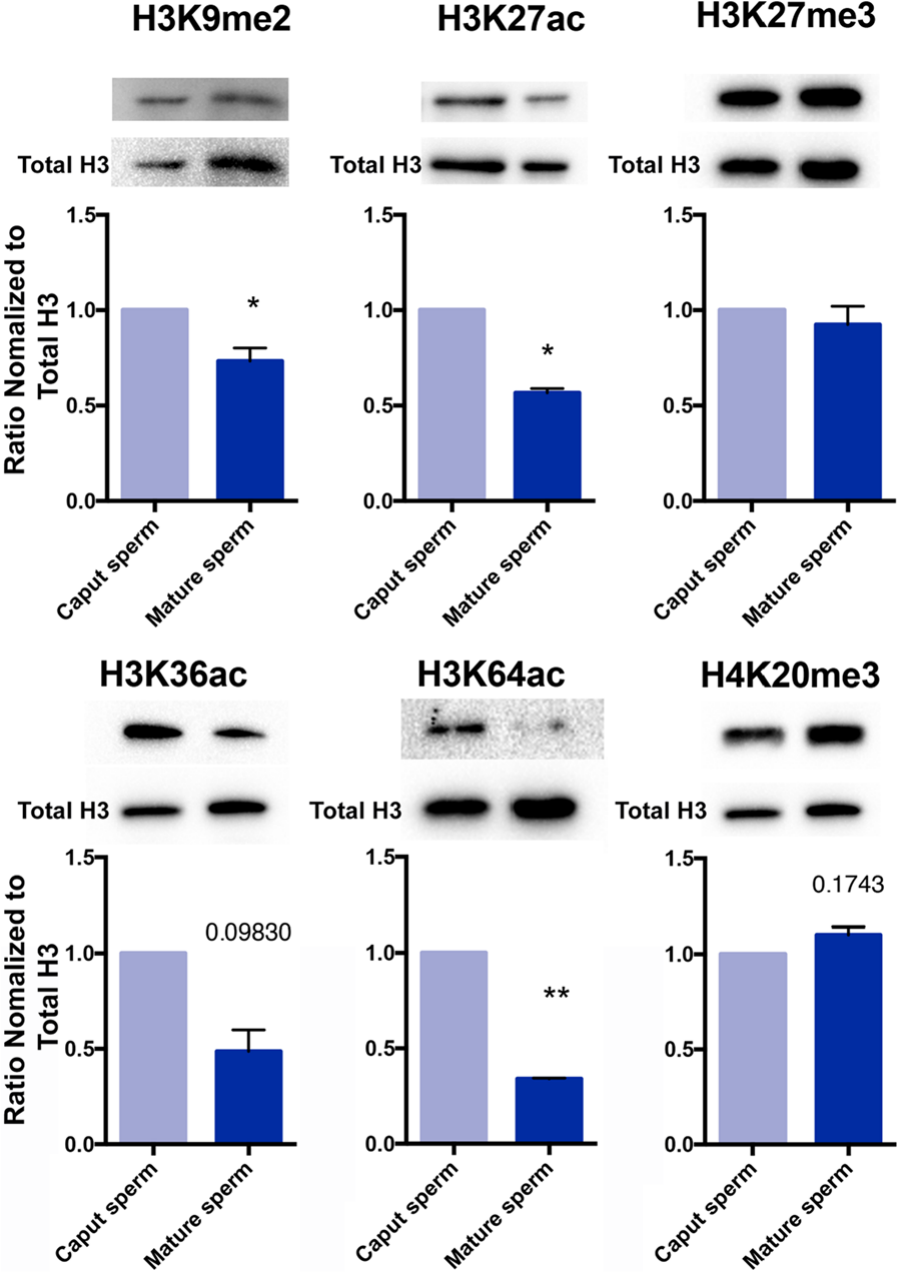
Changes in select histone posttranslational modifications between mouse caput spermatozoa and cauda sperm. Comparison of select histone posttranslational modifications between immature and mature sperm was conducted using Western blotting. Blots of each histone modification were quantified using ImageJ and normalized to levels of total histone H3. This normalized ratio was compared between caput and cauda sperm. Error bars represent the SEM, *n* = 3 independent replicates, * *p* < 0.05, ** *p* < 0.01

To validate the mass spectrometry analysis, we acid-extracted histones from ~ 7.5 million caput and cauda sperm and, using Western blotting, examined changes in six of the posttranslational modifications identified above (Fig. 2). Consistent with the mass-spec analysis, levels of H3K27 trimethylation were identical between caput and cauda sperm, while levels of H3K27 acetylation and H3K9 dimethylation decreased during epididymal maturation (*p* < 0.05). Despite representing a small fraction of the peptide pool, we also confirmed decreased enrichment of H3K64 acetylation between immature and mature sperm. Although decreases in the acetylation of H3K36 and increases in the trimethylation of H4K20 tended to be similar to those observed in our proteomic analysis, these differences did not reach the statistical significance of *p* < 0.05 (specific *p*-values were 0.09830 and 0.1743, respectively). We suspect differences in the sensitivity and variability in Western blotting compared to LC/MS explain these discrepancies [32]. Notably, several posttranslational modifications we validated here are associated with gene enhancer activity. These include histone H3 lysine 27 acetylation (H3K27ac), lysine 64 acetylation, and H3K9 dimethylation (H3K9me2) [33–36]. These observations suggest that gene enhancer-associated posttranslational modifications may be specifically targeted during epididymal transit.

### Alterations in H3K9 dimethylation during epididymal maturation

**Fig. 3:**
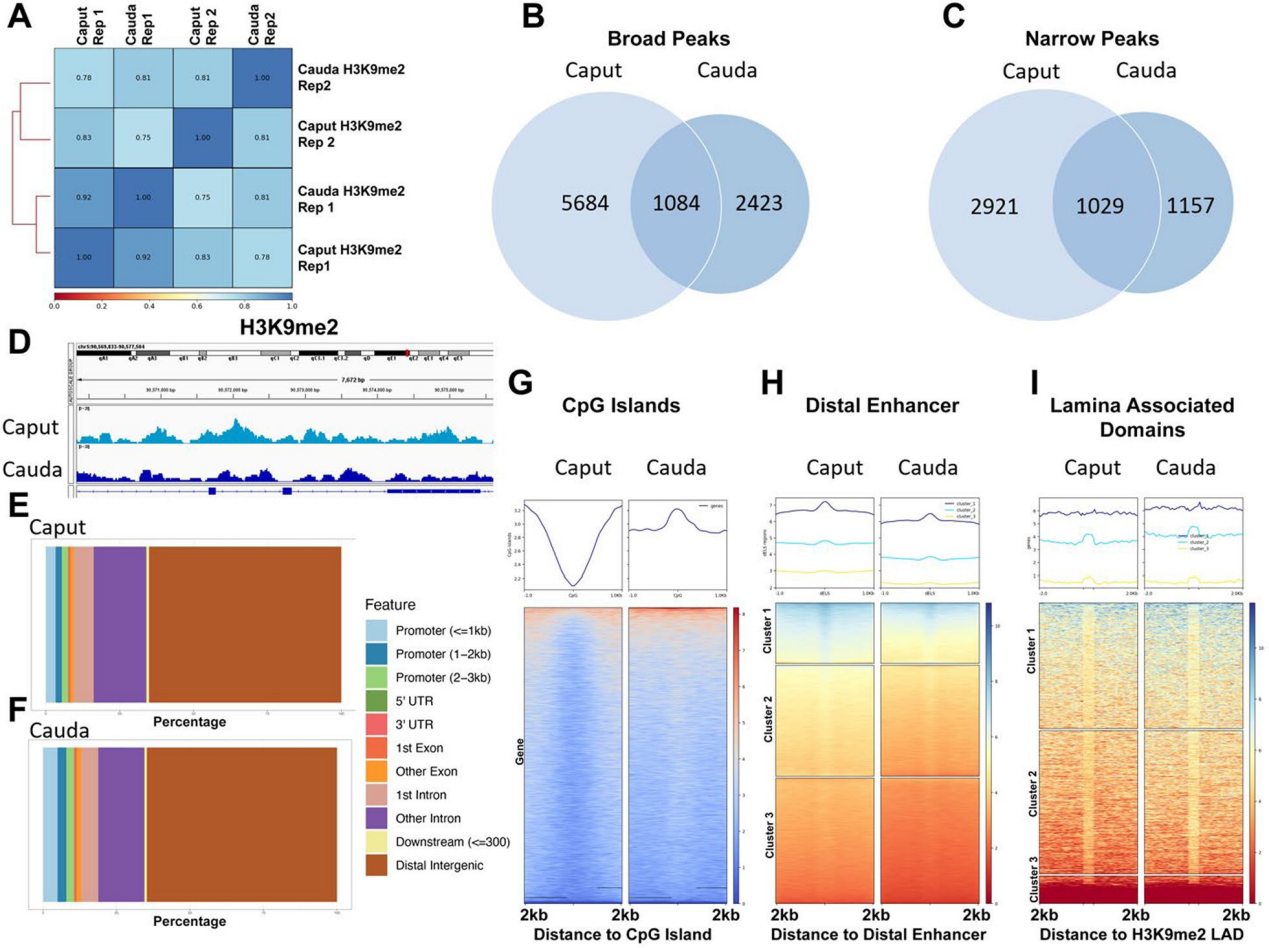
Refinement of sperm H3K9me2 at gene promoters driving spermatogenesis and distal intergenic regions constrained within Lamina-Associated Domains during epididymal maturation. **A** Correlation analysis between H3K9me2 ChIP-seq signals derived from sperm isolated from the caput and cauda regions of the epididymis (*n* = 2). Venn diagrams comparing changes in **B** broad and **C** narrow peaks between caput and cauda-derived sperm. **D** Integrative Genome Viewer tracks of chromosomal regions exhibiting differential H3K9me2 enrichment between caput and cauda-derived sperm. Bar chart representation of the distribution of H3K9me2 across key genomic features between **E** caput and **F** cauda-derived samples. Heatmaps displaying the enrichment of H3K9me2 signals across **G** CpG islands, **H** regions exhibiting a signature of distal enhancers, or **I** Lamina-Associated Domains

To better understand the dynamics of histone structure during epididymal transit, we focused on H3K9me2 and H3K27ac and used chromatin immunoprecipitation followed by deep sequencing to monitor changes in enrichment during sperm maturation. Our analysis of H3K9me2 revealed a strong correlation between biological replicates and between caput and cauda sperm samples (Fig. 3A). Consistent with our LC/MS and Western blot analyses, MACS2 identified decreased enrichment of both broad and narrow peaks between caput and cauda samples, with 6768 broad regions in caput samples decreasing to 3489 broad peaks in cauda (Fig. 3B) and 3950 narrow peaks in caput samples decreasing to 2164 narrow peaks in cauda sperm (Fig. 3C). These changes reflected a general thinning of broad H3K9me2 enrichment into more focused peaks (Fig. 3D). Notably, most H3K9me2-enriched regions mapped to distal intergenic (65%) and intronic regions (25%), consistent between caput and cauda-derived samples (Fig. 3E, F).

Regions enriched in H3K9me2 were generally depleted of CpG islands and did not correlate with gene transcriptional start sites (Fig. 3G). However, when breaking CpG Islands into clusters, we observed that 6% (968 out of 16,014) of Islands displayed an increase in novel H3K9me2 enrichment within cauda-derived samples. Remarkably, most of these regions contained promoter regions with GO pathway analysis identifying enrichment of processes involved in chiasma assembly, synaptonemal complex assembly, chromosome organization, and male gametogenesis (Additional file 2: Figure S1), indicating the promoter regions of genes driving sperm production progressively increase in H3K9me2 during epididymal maturation.

We next examined H3K9me2 enrichment in sequences associated with distal and proximal enhancer-like sequences identified by the ENCODE consortium [37]. Using published ATAC-sequencing profiles [38], we confirmed that a subset of these enhancer-like sequences exhibit an open chromatin conformation in both MII oocytes and sperm (Additional file 2: Figure S2A). In contrast to CpG islands and transcriptional start sites, we observed strong enrichment of H3K9me2 at distal enhancer-like sequences (Fig. 3H). Recent studies indicate that some H3K9me2-enriched regions localize with Lamina-Associated Domains (LADs), which restrain tissue-specific enhancers at the nuclear periphery. Consistent with these studies, GO pathway analysis of novel H3K9me2 regions in cauda-derived sperm identified multiple tissue-specific genes (Additional file 2: Figure S2B). Further, we identified overlap of H3K9me2-enriched regions in caput and caudal sperm with H3K9me2-marked LADs previously identified in ES cells (Fig. 3I) [36]. We conclude from these data that H3K9me2-enriched regions of the sperm genome predominantly map to distal intergenic regions, some of which associate with enhancer-like sequences restrained within LADs.

### Alterations in H3K27 acetylation during epididymal maturation

**Fig. 4:**
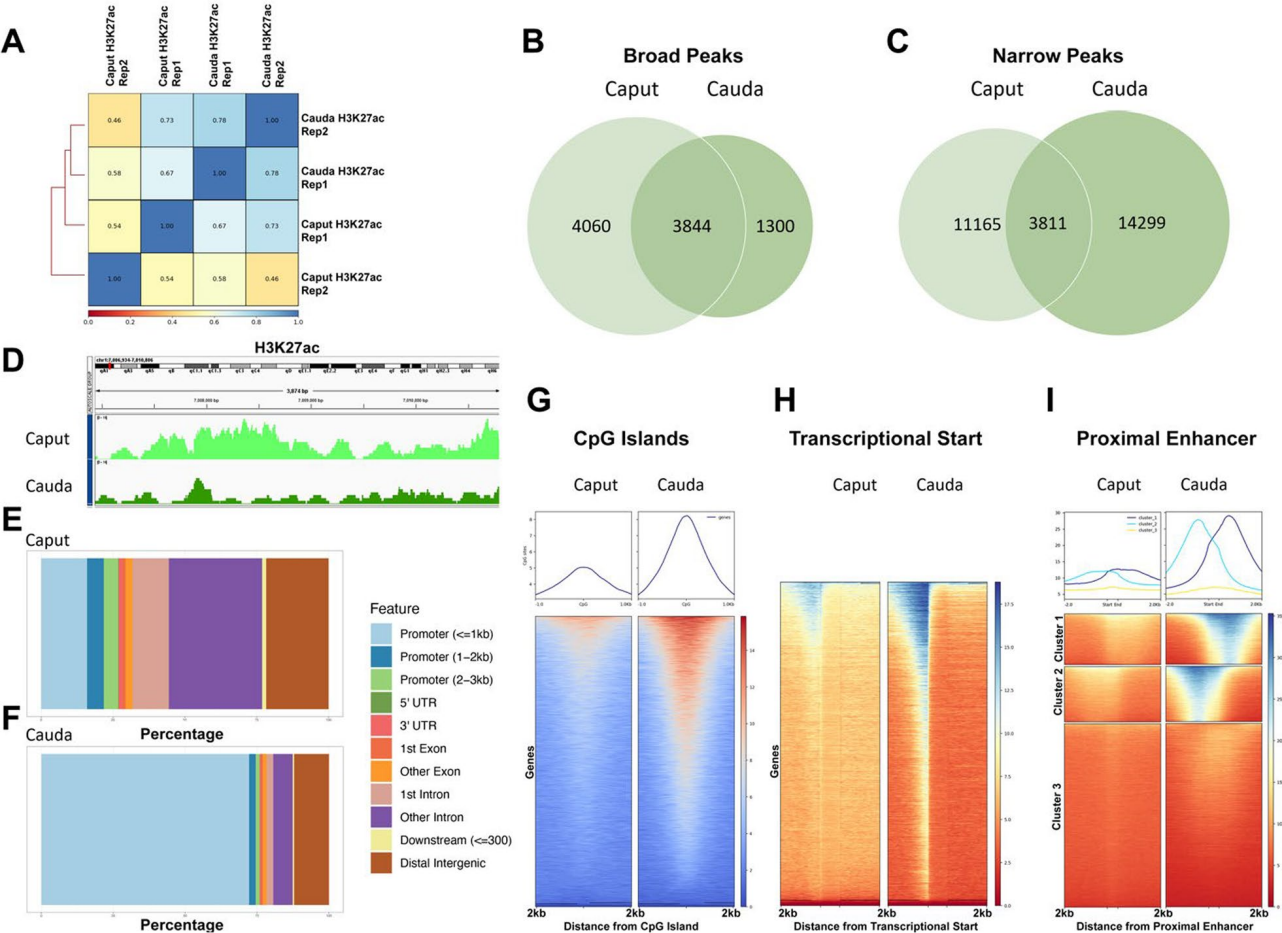
During epididymal maturation, broad H3K27ac peaks sharpen and transition from intronic and distal regions to gene promoters and enhancers driving embryo development. **A** Correlation analysis between H3K27ac ChIP-seq signals derived from sperm isolated from the caput and cauda regions of the epididymis (*n* = 2). Venn diagrams comparing changes in **B** broad and **C** narrow H3K27ac peaks between caput and cauda-derived sperm. **D** Integrative Genome Viewer tracks of chromosomal regions exhibiting the transition from diffuse broad peaks to more focused enrichment between caput and cauda-derived sperm. Bar chart representation of the distribution of H3K27ac across key genomic features between **E** caput and **F** cauda-derived samples. Heatmaps displaying the enrichment of H3K27ac signals across **G** CpG islands, **H** transcriptional start sites, or **I** regions with a proximal enhancer-like signature

Our studies of H3K27ac-enriched regions identified several significant contrasts with the patterns identified for H3K9me2. First, although cauda-derived samples correlated with each other, caput samples were much more variable (Fig. 4A). Second, consistent with H3K9me2, MACS2 identified a reduction in broad peaks (Fig. 4B; caput sperm exhibited 7904 broad peaks, which decreased to 5144 regions in cauda-derived sperm), while in contrast, we identified an increase in narrow peaks (Fig. 4C; 14,976 enriched regions in caput sperm, which increased to 18,110 regions in cauda-derived samples). Interestingly, these changes, again, reflected a sharpening of broad domains into focused peaks (Fig. 4D). Third, unlike H3K9me2, which consistently associated with distal intergenic regions in both caput and cauda-derived sperm, H3K27ac enrichment shifted from distal intergenic (21%) and intronic regions (48%) in caput-derived samples to primarily localizing to gene promoters (72%) and distal intergenic regions (12%) in cauda-derived sperm (Fig. 4E, F). Consistent with this increased enrichment at gene promoters, we identified a significant shift towards the enrichment of H3K27ac on CpG islands (Fig. 4G) and gene transcriptional start sites (Fig. 4H). For the identified gene promoters, pathway analysis identified genetic processes involved in histone modification, embryo development, and multiple aspects of reproductive biology (Additional file 2: Figure S3b).

In addition to gene promoters, we also observed a dramatic sharpening of H3K27ac signals at proximal (Fig. 4H) and distal (Additional file 2: Figure S3a) enhancer-like sequences. For the top 5% of enhancer regions exhibiting increased peak sharpening in cauda-derived sperm (9879 of the 209,041 enhancer regions), pathway analysis identified processes involved in embryo development, pattern specification, embryonic morphogenesis, and CNS development (Additional file 2: Figure S4). Collectively, these observations indicate that during epididymal transit, H3K27 transitions from broad domains to sharp, focused peaks centered on gene promoters and distal enhancers driving early embryonic development. Interestingly, we observed a weak negative correlation between H3K9me2 and H3K27ac, suggesting these marks may oppose each other (Additional file 2: Figure S5).

### Broad domains of H3K27ac in sperm partially overlap with broad domains of H3K4me3 in MII oocytes

**Fig. 5:**
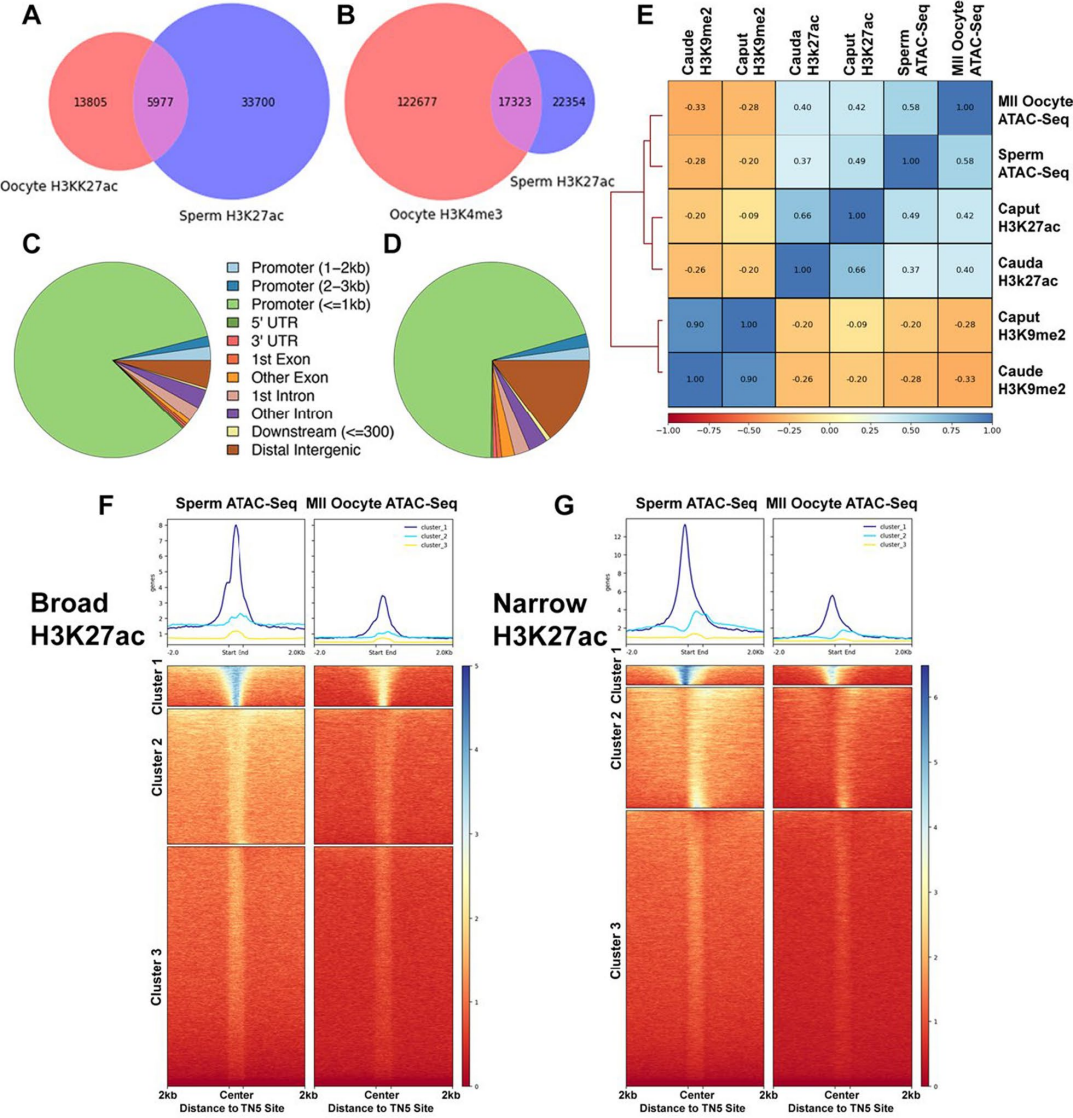
Broad domains of H3K27ac in caudal sperm overlap with broad domains of H3K4me3 in MII Oocytes and colocalize to regions of open chromatin identified in the ATAC-seq profiles of MII oocytes and sperm. Venn diagrams comparing broad domains of H3K27ac identified in caudal sperm to broad domains of H3K27ac (**A**) and H3K4me3 (**B**) previously identified in MII oocytes (dataset GSE72784, [39]). Overlapping H3K27ac-H3K27ac (**C**) and H3K4me3-H3K27 (**D**) regions predominantly map to within 1 kb of gene promoters. **E** Correlation analysis between H3K9me2 and H3K27ac-enriched regions in caput and caudal sperm with regions of open chromatin identified using ATAC-seq in MII oocytes and sperm (GSE116854 [38]). Heatmaps showing the localization of broad (**F**) and narrow (**G**) H3K27ac peaks in caudal sperm at ATAC-seq Tn5-hypersensitive sites sperm and MII oocytes

The gametic transmission of information organizing embryonic chromatin states is complex and likely involves multiple factors. Previous studies examining chromatin profiles of MII oocytes and sperm identified sets of both common and gamete-specific Tn5-hypersensitive sites, signifying open chromatin conformations [38]. In oocytes, regions with accessible chromatin correlate with broad domains of H3K4me [39]. Therefore, we examined the alignment of the H3K27ac-enriched broad domains we identified in caudal sperm with broad domains of H3K4me3 and H3K27ac previously identified in oocytes (dataset GSE72784, [39]). Despite a lower overall enrichment of H3K27ac, caudal sperm contain a greater abundance of H3K27ac-enriched broad domains than MII oocytes (Fig. 5A). We identified 15% overlapping H3K27ac broad domains shared between gametes, which overwhelmingly mapped to within 1 kb of gene promoters (Fig. 5C). Interestingly, when we compared broad domains of H3K4me3 in oocytes to broad domains of H3K27ac in caudal sperm, we observed a more significant overlap, with 17,323 peaks of the 39,677 (44%) regions identified in caudal sperm overlapping (Fig. 5B). These regions predominantly map to within 1 kb of the promoters of approximately 13,000 genes (Fig. 5D), enriched in processes relating to ribosomal biogenesis and RNA metabolism. A comparison of H3K27ac-enriched regions in caput and caudal sperm with Tn5-hypersensitive sites in both oocytes and sperm [19, 38] revealed a positive correlation, while H3K9me2-enriched regions displayed a negative correlation (Fig. 5E). Using Kmeans clustering, we identified strong enrichment of H3K27ac-enriched broad, and to a lesser extent, narrow domains over a subset of Tn5-hypersensitive sites in MII oocytes and sperm (Fig. 5F, G). Again, genes in the top clusters are enriched in functions related to embryo development, and organismal survival. We did not observe any significant overlap of H3K9me2 broad or narrow peaks with Tn5-hypersensitive sites in either gamete.

### Histone deacetylase expression in sperm isolated from the head caput and caudal portions of the mouse epididymis

**Fig. 6:**
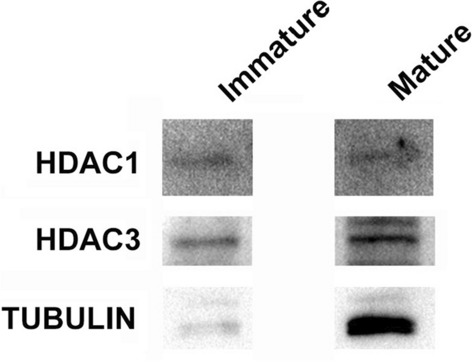
Identification of Class I histone deacetylase enzymes HDAC1 and HDAC3 in mouse sperm. Expression of HDAC1 and HDAC3 was assayed in immature and mature sperm using Western blotting. We derived protein extracts from 7.5 million caput spermatozoa and 38 million cauda sperm. Note: we cannot quantitatively compare bands between immature and mature sperm as we loaded unequal amounts of protein

In somatic cells, the histone deacetylase family of enzymes removes acetyl groups from lysine residues on histone tails. This family is divided into three classes based on their exclusive localization to the nucleus (Class I), their ability to shuttle between the cytoplasm and the nucleus (Class II), and if they require the cofactor NAD + for deacetylase activity (Class III) [40]. Of these three, Class I deacetylases frequently associate with transcription factors and drive the decommissioning of gene enhancers [41, 42]. Given that most changes identified by our LC/MS analysis involve histone deacetylation, we examined sperm for the presence of Class I HDACs. Using RIPA buffer, we isolated protein extracts from ~ 7.5 million caput and cauda sperm, then examined the expression of HDAC1, HDAC2, and HDAC3 using Western blotting. We detected both HDAC1 and HDAC3 in spermatozoa isolated from the caput portion of the epididymis (Fig. 6). When we increased the number of mature sperm fivefold to 38 million (isolated from four mice), we detected both HDAC1 and HDAC3 in mature sperm (Fig. 6). We were unable to detect HDAC2 in any sperm samples. From these data, we conclude that HDAC1 and HDAC3 are present in both immature and mature mouse sperm.

### Differences in histone posttranslational modifications during epididymal maturation of ram sperm

**Fig. 7:**
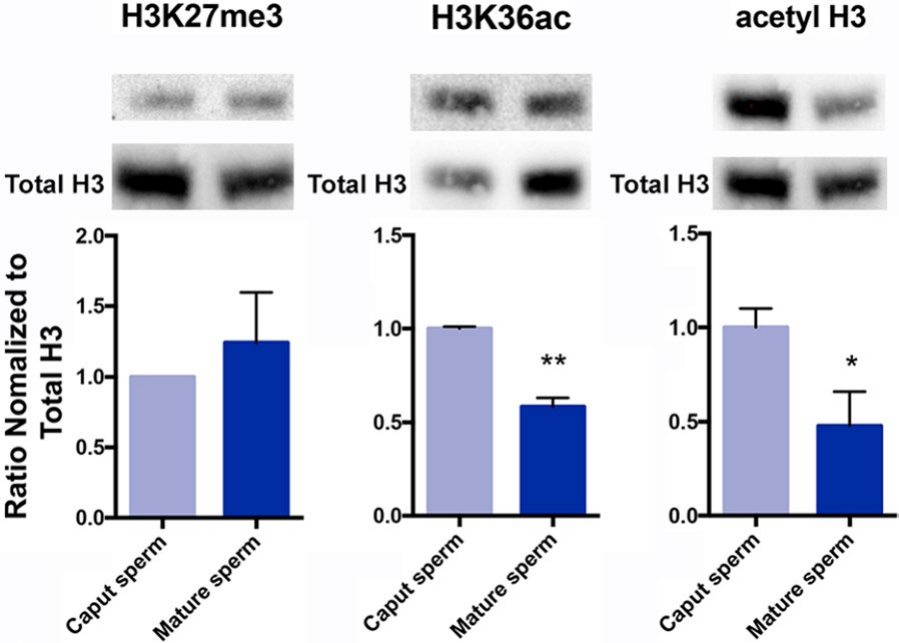
Changes in histone posttranslational modifications during the epididymal maturation of ram sperm. Comparison of histone posttranslational modifications between immature and mature sperm using Western blotting. Blots of each histone modification were quantified using ImageJ and normalized to levels of total histone H3. This normalized ratio was compared between caput and cauda sperm. Error bars represent the SEM, *n* = 3 males, * *p* < 0.05, ** *p* < 0.01

Finally, we sought to determine if changes in histone posttranslational modifications are a common feature of mammalian epididymal maturation. To this end, we assayed patterns of histone structure between immature and mature sperm isolated from *Ovis aries* (Rambouillet Merino). Similar to mouse sperm, less than 5% of the ruminant genome retains histones [43], and both species exhibit an extended (10 to 14 days) epididymal transit time compared to humans [44]. After euthanasia, we dissected the ram reproductive tract and separately isolated sperm from the caput and caudal regions of the epididymis. We then assayed changes in histone structure between caput spermatozoa and cauda sperm using Western blotting. Similar to mice, we did not observe any significant changes in the levels of H3K27me3 between caput spermatozoa and mature sperm, while levels of H3K36ac diminished during epididymal transit (Fig. 7). In addition, although blots using H3K27ac and H3K64ac antibodies failed to identify appropriately sized bands for histone H3 (data not shown), we detected a significant reduction in pan-histone H3 acetylation between caput spermatozoa and caudal sperm. These data indicate that, in addition to mice, rams also exhibit changes in histone posttranslational modifications during epididymal maturation.

## Discussion

This study's core finding is that chromatin structure changes during epididymal transit and that these alterations occur on sequences containing gene promoters, as well as both proximal and distal regulatory elements. Previous studies examining chromatin structure in mature sperm have primarily focused on modifications associated with bivalent gene promoters [20]. Our study did not observe differences in either H3K4me3 or H3K27me3, suggesting that bivalent chromatin is stable during epididymal maturation. Significantly, with the exception of increased H4K16ac, our LC/MS comparisons of caput spermatozoa and cauda sperm identified changes in all histone posttranslational modifications associated with enhancer activity characterized to date [33–36, 45–47], as well as selective increases in modifications hypothesized to oppose enhancer chromatin topology [48]. Specifically, acetylation of H4K16 maintains an open state by impeding the ability of chromatin to form cross-fiber interactions and is directly opposed by methylated histone H4K20 [49, 50]. Both modifications are in the top 12 differentially enriched posttranslational modifications. Future studies will determine if these modifications colocalize with gene regulatory elements.

During the transition from the oocyte to 2-cell embryo, H3K4me3 enrichment remodels from broad, non-canonical domains into narrow peaks, and this change is essential to the initiation of transcription during zygotic genome activation [39, 51, 52]. Here, we observe a similar transition from broad to narrow peaks for H3K9me2 and H3K27ac, with cauda H3K27ac-enriched regions also displaying a pronounced shift from gene bodies to the promoters and enhancer regions of genes driving embryonic development. Interestingly, some H3K27 broad domains are shared with broad H3K4me3 regions in oocytes and localize to regions of open chromatin identified in both oocytes and sperm [19, 38]. The focused enrichment of these regions on gene promoters suggests that broad domains of H3K4me3 in oocytes and H3K27ac in sperm may demarcate genes required for the earliest phase of embryonic development.

In contrast to H3K27ac, gene promoters associated with meiosis and sperm production, as well as multiple tissue-specific gene enhancers, displayed increased enrichment of H3K9me2, a modification associated with repressed chromatin states held at the nuclear periphery [36, 53, 54]. Importantly, recent studies reveal an evolutionally conserved role for H3K9me2 in transmitting the three-dimensional structure of the genome through mitosis [54]. Our observations indicate that many H3K9me2-enriched LAD regions are also present in sperm and are similarly enriched with tissue-specific enhancers.

One limitation to the current study is that our LC/MS analysis is biased to measure changes on histone H3 and has a limited ability to identify histone variants. Therefore, we may have missed changes to residues on other histones or specific histone variants. Importantly, we only observed reductions in lysine 27 acetylation on the histone H3.3 variant and not on H3.1/3.2. Histone H3.3 specifically incorporates into gene promoter and enhancer regions and impedes intrafiber folding, maintaining the open conformation necessary to bind cofactors required to activate transcription [55]. Significantly, the H3.3 variant persists through the transition from the sperm genome to the paternal pronucleus after fertilization [56]. The selective targeting of histone H3.3 in maturing sperm further supports the H3 barcode hypothesis, positing that region-specific incorporation of histone variants serves as a backup for less stable posttranslational modifications and represents the first layer of epigenetic memory [57]. We suspect that the ability of H3.3 to maintain an open conformation allows the selective targeting of H3.3-enriched loci in the compacted sperm nucleus, a process that may be influenced by noncoding RNAs [58]. Class I HDACs complex with lysine-specific demethylase 1 (also known as KDM1A) to demethylate histone H3 on Lys 4 and Lys 9 (H3K4/K9). During stem cell differentiation, the cooperative interaction of these chromatin modifiers within the nucleosome remodeling and histone deacetylase (NuRD) complex is essential for decommissioning enhancers driving pluripotency [42]. Although not conclusive, identifying HDAC1 and HDAC3 here in mouse sperm and by others in human sperm [59] suggests a similar process may occur during epididymal maturation. Alternatively, the open confirmation conferred by histone variant H3.3 may predispose enhancer regions to protamine replacement, which recent studies suggest continues during epididymal maturation [12].

## Conclusion

Collectively, our data indicate that epididymal maturation includes the refinement and focusing of two essential posttranslational histone modifications that further hone the paternally inherited epigenome and may transmit information on the spatial organization of the genome to the early embryo.

## Materials and methods

### Mouse sperm isolation and histone acid extraction

Experiments involving mice were conducted under AUP 2017-0308 and approved by the Texas A&M University IACUC. We obtained male C57BL/6N mice (RRID:IMSR_JAX:005304) from the Texas A&M Institute for Genomic Medicine and maintained them on a standard diet (catalog# 2019, Teklad Diets, Madison, WI, USA) with a 12-h light/dark cycle. We dissected the male reproductive tract from adult (< postnatal day 90) males and separately placed the initial segment of the caput and the entire portion of the cauda, plus approximately 1 cm of vas deferens, into individual wells containing 500 μL of pre-warmed (37 °C) Human Tubal Fluid medium (catalog# ZHTF-100, Zenith Biotech, Blue Bell, PA, USA). Next, we made four or five incisions to each separated section of the epididymis to allow sperm to swim out and used forceps to extrude sperm from the 1 cm portion of the vas deferens. Next, we incubated plates at 37 °C for 30 min, collected sperm, and diluted a 10-μl aliquot 1:50 in diH2O to count cells using a Neubauer chamber slide. We washed samples in PBS, then incubated sperm in somatic cell lysis buffer (0.1% SDS, 0.5% Triton- X-100) for 30 min on ice. We confirmed purity using microscopy, centrifuged and washed the samples in PBS, then snap-froze sperm pellets and stored them at − 80 °C.

We acid-extracted histones using a modified version of the previously described procedure [60]. We resuspended frozen sperm pellets in Nuclei Isolation Buffer-250 with 0.3% NP-40 (15 mM Tris–HCl (pH 7.5), 60 mM KCl, 15 mM NaCl, 5 mM MgCl2, 1 mM CaCl2, 250 mM sucrose, 1 mM DTT, 10 mM sodium butyrate, and 1:100 Halt protease inhibitor (Cat# PI78437, Thermo Fisher Scientific, Pittsburgh, PA, USA)) and rotated them at 4 °C for 30 min. We verified sperm lysis using microscopy and then centrifuged samples at 600 × g for five minutes. We washed the samples twice using Nuclei Isolation Buffer-250 without NP40, resuspended the pellet in five volumes of 0.2 M H2SO4, and rotated the samples overnight at 4 °C. We then centrifuged samples at 4,000 × g for 4 min and transferred the histone-enriched supernatant into new tubes. We added trichloroacetic acid to a final concentration of 20% by volume, then incubated samples for 2 h on ice. We centrifuged samples at 10,000×g for 5 min at 4 °C, discarded the supernatant, and resuspended the pellet in 1 mL cold acetone/0.1% HCl. We then washed the pellet twice with 100% acetone, air-dried the sample, and resuspended the pellet in water.

### Ram sperm isolation and histone acid extraction

Experiments utilizing rams were conducted under AUP 2017–0210 and approved by the Texas A&M University IACUC. After euthanasia, we isolated the reproductive tract from 14-month-old Rambouillet Merino males. We made large incisions across the proximal and distal loops of the caput and the entire cauda, then separately placed the caput and cauda into 50-ml conical tubes filled with warmed PBS. We then incubated the tubes in a 37 °C water bath for 30 min to allow sperm to swim out. We washed sperm twice in fresh PBS, then incubated the sperm in somatic cell lysis buffer (0.1% SDS, 0.5% Triton X-100) for 30 min on ice. We washed cells in PBS, incubated sperm in 50 mM DTT for 30 min at room temperature, then sonicated cells for 5 min (five 30-s pulses) using a Bioruptor sonication system (Diagenode, Denville, NJ, USA). We pelleted cells by centrifugation, resuspended the pellet in Nuclei Isolation Buffer-250 without NP40, and incubated the samples at 4 °C with constant rotation for 30 min. We pelleted cells by centrifugation at 600×g, resuspended the pellet in 0.2 M H2SO4, sonicated the samples again for five minutes (five 30-s pulses), and then incubated the samples overnight at 4 °C with constant rotation. We then added trichloroacetic acid to the supernatant with a final concentration of 20% by volume and incubated samples on ice for two hours. We centrifuged samples at 10,000×g for five minutes at 4 °C, discarded the supernatant, and washed the pellet twice in ice-cold acetone. We let the samples air dry for 20 min and then resuspended the pellet in water.

### Mass spectrometry

We assayed the profile of sperm histones using the ModSPec service from Active Motif (Carlsbad, CA, USA). Pelleted histone peptides were resuspended in 0.1% TFA in water and analyzed on a TSQ Quantiva triple quadrupole (QqQ) mass spectrometer directly coupled with an UltiMate 3000 Dionex nano-liquid chromatography system (Thermo Fisher Scientific, Pittsburgh, PA, USA). Peptides were first loaded onto an in-house packed trapping column (3 cm × 150 μm) and then separated on a New Objectives PicoChip analytical column (10 cm × 75 μm). Both columns were packed with New Objectives ProntoSIL C18-AQ, 3 μm, 200 Å resin. The chromatography gradient was achieved by increasing the percentage of buffer B from 0 to 35% at a flow rate of 0.30 μl/min over 45 min (Solvent A: 0.1% formic acid in water, and Solvent B: 0.1% formic acid in 95% acetonitrile). The QqQ settings were as follows: collision gas pressure of 1.5 mTorr; Q1 peak width of 0.7 (FWHM); cycle time of 2 s; skimmer offset of 10 V; electrospray voltage of 2.5 kV. Targeted analysis of unmodified and various modified histone peptides was performed. This entire process was repeated three separate times for each sample.

### Total protein isolation and Western blotting

We homogenized fresh sperm in Tris lysis buffer (50 mM Tris, 1 mM EGTA, 150 mM NaCl, 1% Triton X-100, 1% 2-mercaptoethanol, 50 mM NaF, 1 mM Na3VO4; at pH 7.5). We separated protein extracts (either sperm total protein or acid-extracted histones) on 10% sodium dodecyl sulfate polyacrylamide gels and transferred proteins to PVDF membranes. Blots represent pooled caput spermatozoa collected from eight to ten males, while cauda sperm consisted of mature sperm isolated from one or the pooled sperm from two males. The primary antibodies we used in this study are as follows: antiH3K9me2 (Cat# 39240, Active Motif Carlsbad, CA, USA), antiH3K27me3 (Cat# 07–449; RRID:AB_310624; Millipore-Sigma, St. Louis, MO, USA), antiH3K27ac (cat# ab4729; RRID:AB_2118291; Abcam, Cambridge, MA, USA), antiH3K64ac (Cat# ab214808, Abcam) antiH3K36ac (Cat# ab177179, Abcam), antiH4K20me3 (Cat# ab9053; RRID:AB_306969; Abcam), anti-panacetyl-Histone H3 (Cat# 06-599; RRID:AB_2115283; Millipore-Sigma) and antiH3 (Cat# ab1791; RRID:AB_302613; Abcam). We visualized blots using secondary antibodies conjugated to horseradish peroxidase (catalog no. sc-2004; RRID:AB_631746; Santa Cruz Biotechnology, Santa Cruz, CA, USA) and an enhanced chemiluminescence detection system (LI-COR, Lincoln, Nebraska, USA). The data presented in Fig. 6 only assay the presence of HDAC1 and HDAC3, and as we loaded unequal amounts of protein, they are not quantitative.

### Sperm chromatin immunoprecipitation sequencing (ChIP-seq)

We carried out sperm histone ChIP using a previously published protocol [61]. Briefly, for each biological rep, sperm collected from the caputs and cauda of 10–15 mice were pooled, purified, and pre-treated with 50 μM DTT for 2 h at RT to open sperm chromatin. Cell suspensions were split into roughly 15 million sperm aliquots per IP and treated with 90 U (12 U per 2 million sperm) of MNase (New England Biolabs, #M0247S) for 5 min at 37C. MNase-digested chromatin was then pre-cleared with blocked A/G Sepharose beads for 1 h at 4C. For IP, 5 μg of antibody (H3K27ac or H3K9me2) was added to the pre-cleared chromatin overnight, followed by 50 μL of blocked beads for 4 h at 4C. Next, we washed the beads and then eluted DNA using standard elution buffer. Following RNase A and proteinase K treatment, we purified DNA using phenol/chloroform isolation and ethanol precipitation and resuspended it in 40 μL ultrapure water.

Library preparation using 10 ng of DNA and 75 bp paired-end sequencing with at least 50 million reads per sample was conducted by the UTHealth Cancer Genomics Core. Raw fastq reads were trimmed for adapters and quality and then mapped to the mm10 reference genome using BWA-MEM [62].

### Data analysis

#### Mass-spec analysis

Raw mass-spec files were imported and analyzed in Skyline with Savitzky–Golay smoothing [63]. All Skyline peak area assignments for monitored peptide transitions were manually confirmed. A minimum of 3 peptide transitions were quantified for each modification. We quantified the modified and unmodified forms for each monitored amino acid residue by calculating the sum of peak areas of all corresponding peptide transitions and the unmodified forms. We then determined the percentage of each modification of the total sum of unmodified and modified forms. This process was carried out for each of the three separate LC/MS runs. We then imported the raw data into Excel to calculate the mean and standard deviation for each modified and unmodified form of the corresponding amino acid residue.

#### Western blot analysis

For the analysis of Western blots, we quantified band intensities using the densitometry feature of ImageJ (RRID:SCR_003070; National Institutes of Health, Bethesda, MD, USA) and, after importing the obtained values into Excel, derived a ratio of the intensity of the modified histone divided by the intensity for total histone H3. We imported these ratios into the statistical analysis program GraphPad (RRID:SCR_002798; GraphPad Software, Inc., La Jolla, CA, USA) and set statistical significance at alpha = 0.05. Finally, we verified all datasets for normality using the Brown-Forsythe test, then compared histone ratios using an unpaired student’s t-test.

#### ChIP-seq analysis

We uploaded all sequence files to the Galaxy server [64] (usegalaxy.org) for further processing. We then used Trimmomatic [65] to trim FASTQ files for end quality and remove adapters. We mapped trimmed reads to the mouse reference genome (mm10) using the Burrows-Wheeler Alignment tool BWA [62] and only retained reads with a minimum MAPQ score of 20 for downstream analysis as BAM files. We downloaded published oocyte H3K4me3 and H3K27ac ChIP-seq profiles (Geo: GSE72784, [39]) and processed reads using the same workflow.

#### Peak calling and differential peak analysis

We used MACS2 [66] to analyze ChIP-seq datasets and to call both broad and narrow peaks enriched against mapped reads obtained from Input files with an FDR threshold of 0.1. We determined the fragment size ‘d’ from alignment results using the MACS2 predictd tool, and we used this data as the extension size for the MACS2 callpeak tool. We converted Bedgraph treatment file outputs to BigWig files for visualization in Integrative Genomics Viewer (IGV) [67]. We then extracted peaks from the broadpeaks and narrowpeaks outputs common to all Control samples and all Treatment samples using BEDTools_intersect_intervals [68]. We only considered intersecting peaks if at least a 0.25 fraction of the genomic intervals overlapped in both sequencing runs. We used ChIPSeeker [69] to map these overlapping regions. We downloaded published sperm and oocyte ATAC-seq profiles from Geo: GSE116854 [38] and used CrossMap [70] to move onto the mm10 genome. We then compared the converted BigWig files to various genomic regions of interest using features using deepTools2 [71].

#### Visualization of peaks and regions of interest

We converted all peak files to BigWig files and visualized the separate tracks in IGV. We then loaded the BED files marking genomic intervals of peaks gained or lost directly from Galaxy to IGV. We obtained BED files containing chromosomal coordinates for CpG islands and gene bodies from UCSC. We downloaded the BED file (accession ID ENCSR695LYW), demarcating candidate cis-regulatory elements with distal and proximal enhancer-like signatures for mm10, from the ENCODE portal (https://www.encodeproject.org/) [37]. Finally, we obtained sequences for putative H3K9me2-enriched Lamina-Associated Domains from previous studies in mouse ES cells [36] and contrasted our datasets using deepTools2 [71].

## Supplementary Information

**Additional file 1: Table S1**. List of all histone posttranslational modifications examined using nano-liquid chromatography followed by triple quadrupole mass spectrometry (LC/MS). Each posttranslational modification is presented as a percentage of the total peptide pool, where the total pool is the sum of unmodified and modified forms.

**Additional file 2: Figure S1–S5.** Genomic features and results of Pathway Analysis for the enriched peaks identified in Figs. 3 and 4.

## Data Availability

The datasets generated and/or analyzed during the current study are available in the Gene Expression Omnibus (GEO) repository under the series record GSE185603.

## Abbreviations

LC/MS: Nano-liquid chromatography followed by triple quadrupole mass spectrometry
LADs: Lamina-Associated Domains
HDAC1: Histone deacetylase 1
HDAC2: Histone deacetylase 2
HDAC3: Histone deacetylase 3
KDM1A: Lysine-specific demethylase 1
NuRD: Nucleosome remodeling and histone deacetylase complex

## Footnotes

1 Modifications to histone H3 or H4 are abbreviated as histone H3 or H4, followed by the position of the specific lysine residue (K#) and finally, the specific modification, where UN = unmodified, me1 monomethylation, me2 = dimethylation, me3 = trimethylation and ac = acetylation.
