# Supplementary material for: Chromatin alterations during the epididymal maturation of mouse sperm refine the paternally inherited epigenome
Source: Epigenetics Chromatin. 2022 Jan 6;15:2. doi: 10.1186/s13072-021-00433-4 (PMC8734183; doi:10.1186/s13072-021-00433-4)
Supplement: Supplementary file 2 — Additional file 2: Figure S1–S5. Genomic features and results of Pathway Analysis for the enriched peaks identified in Figs. 3 and 4. [file 13072_2021_433_MOESM2_ESM.pdf]

Supplemental Figure 1.
Genomic distribution (top) and top 25 Go Pathways (below) for Cluster 1 CpG Islands that display an increase in novel H3K9me2 enrichment within cauda-derived sperm.

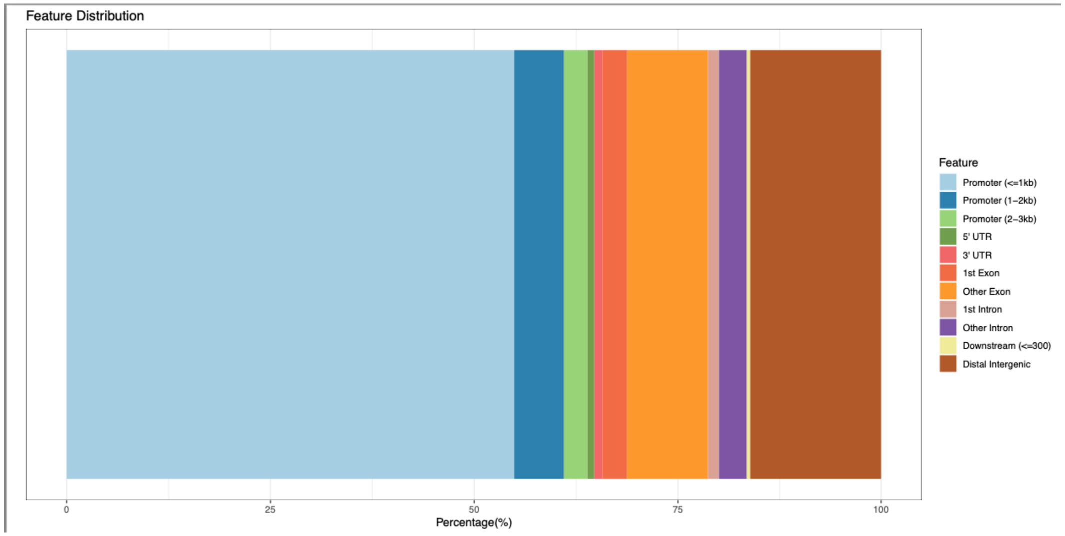

Analysis Type:	PANTHER Overrepresentation Test (Released 20210224)
Annotation Version and Release Date:	GO Ontology database DOI: 10.5281/zenodo.4735677 Released 2021-05-01
Analyzed List:	upload_1 (Mus musculus)
Reference List:	Mus musculus (all genes in database)
Test Type:	FISHER
Correction:	FDR

GO biological process complete	Mus musculus - REFLIST (21988)	#	expected	+/-	Fold Enrichment	raw P value	FDR
chiasma assembly (GO:0051026)	7	4	0.15	+	26.18	6.03E-05	1.38E-02

cell cycle phase (GO:0022403)	21	6	0.46	+	13.09	1.86E-05	6.12E-03
synaptonemal complex assembly (GO:0007130)	24	6	0.52	+	11.45	3.54E-05	8.72E-03
synaptonemal complex organization (GO:0070193)	27	6	0.59	+	10.18	6.25E-05	1.41E-02
calcium-dependent cell-cell adhesion via plasma membrane cell adhesion molecules (GO:0016339)	34	7	0.74	+	9.43	2.33E-05	6.45E-03
biological phase (GO:0044848)	38	7	0.83	+	8.44	4.37E-05	1.04E-02
homologous chromosome pairing at meiosis (GO:0007129)	57	10	1.24	+	8.04	1.50E-06	1.13E-03
homologous chromosome segregation (GO:0045143)	71	11	1.55	+	7.1	1.35E-06	1.12E-03
chromosome organization involved in meiotic cell cycle (GO:0070192)	80	11	1.75	+	6.3	3.83E-06	2.02E-03
synapse assembly (GO:0007416)	86	11	1.88	+	5.86	7.18E-06	3.15E-03
positive regulation of synapse assembly (GO:0051965)	80	10	1.75	+	5.73	2.24E-05	6.79E-03
meiosis I (GO:0007127)	125	15	2.73	+	5.5	3.37E-07	4.83E-04
homophilic cell adhesion via plasma membrane adhesion molecules (GO:0007156)	109	13	2.38	+	5.46	2.17E-06	1.49E-03
meiosis I cell cycle process (GO:0061982)	127	15	2.77	+	5.41	4.05E-07	5.33E-04
meiotic chromosome segregation (GO:0045132)	100	11	2.18	+	5.04	2.62E-05	6.76E-03
multicellular organismal response to stress (GO:0033555)	94	10	2.05	+	4.87	7.84E-05	1.72E-02
cell-cell adhesion via plasma-membrane adhesion molecules (GO:0098742)	190	19	4.15	+	4.58	1.31E-07	2.30E-04
regulation of synapse assembly (GO:0051963)	121	12	2.64	+	4.54	2.92E-05	7.43E-03

positive regulation of cell junction assembly (GO:1901890)	123	12	2.69	+	4.47	3.39E-05	8.49E-03
meiotic nuclear division (GO:0140013)	169	16	3.69	+	4.34	2.48E-06	1.50E-03
meiotic cell cycle process (GO:1903046)	185	16	4.04	+	3.96	7.23E-06	3.08E-03
central nervous system neuron differentiation (GO:0021953)	215	17	4.69	+	3.62	1.13E-05	4.15E-03
regulation of synapse organization (GO:0050807)	261	19	5.7	+	3.33	1.05E-05	3.95E-03
cell junction assembly (GO:0034329)	249	18	5.44	+	3.31	1.95E-05	6.16E-03
nuclear chromosome segregation (GO:0098813)	212	15	4.63	+	3.24	1.19E-04	2.46E-02
meiotic cell cycle (GO:0051321)	299	21	6.53	+	3.22	6.11E-06	2.83E-03

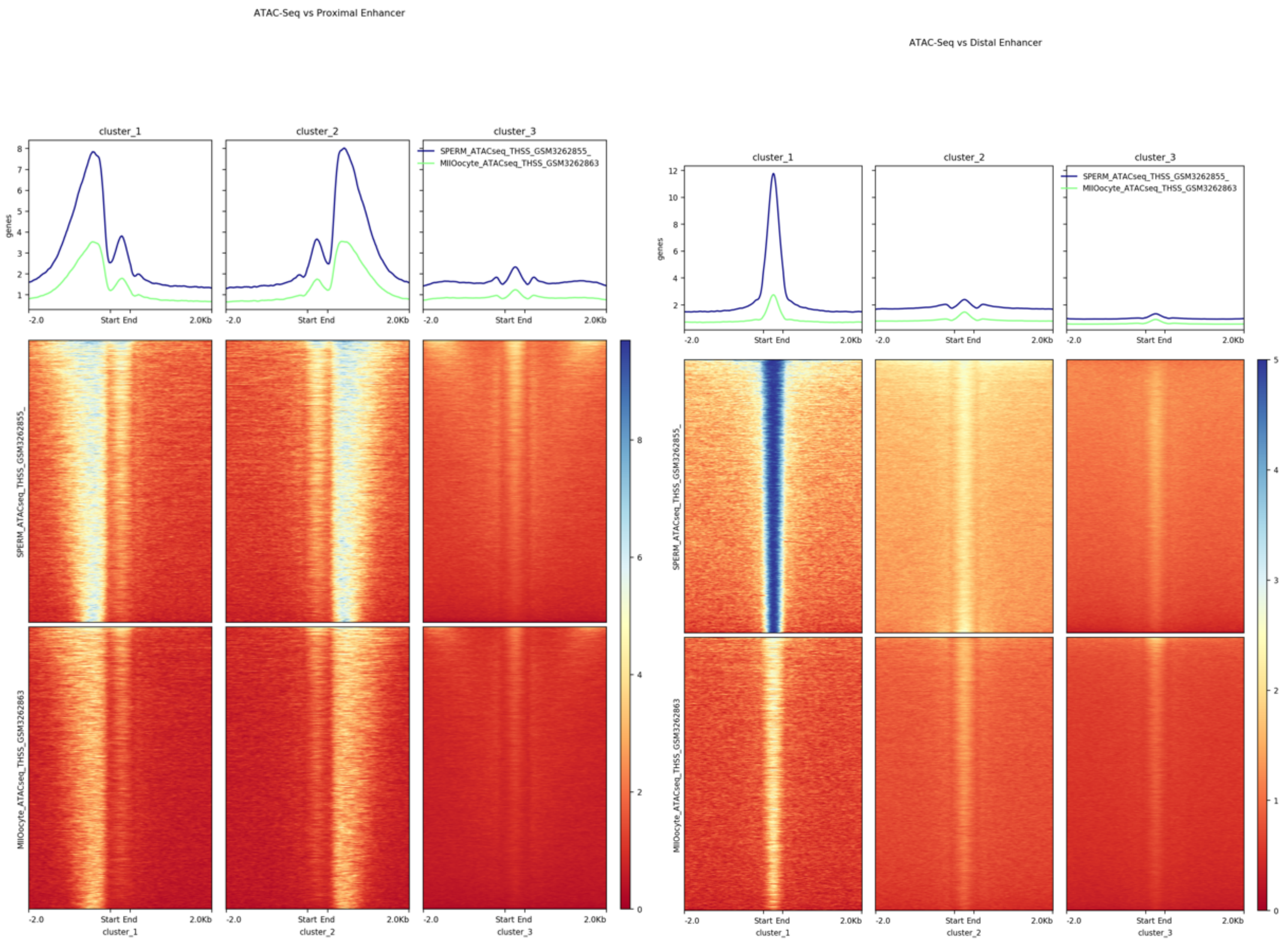

Supplemental Figure 2A. ATAC-sequencing profiles compared to Distal and Proximal Enhancers. Using deepTool2, we compared Tn5 transposase hypersensitive sites identified in MII oocytes and caudal sperm (Geo: GSE116854) to distal and proximal enhancer-like sequences identified by the ENCODE consortium (37).

Supplemental Figure 2B. Genomic distribution (top) and top 25 Go Pathways (below) for Cluster 1, Enhancer-Like Sequences exhibiting increased H3K9me2 enrichment in cauda-derived sperm.

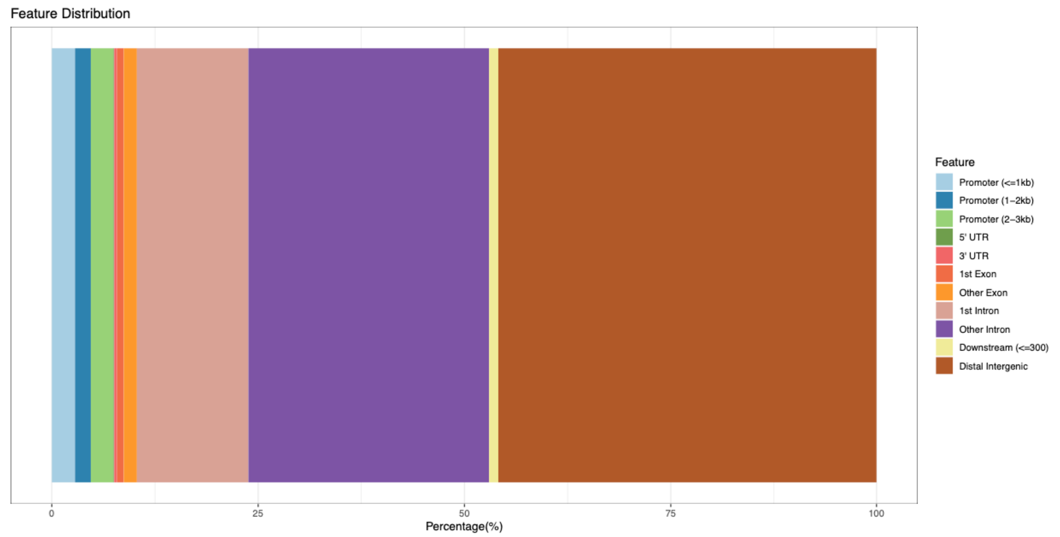

Analysis Type:	PANTHER Overrepresentation Test (Released 20210224)
Annotation Version and Release Date:	GO Ontology database DOI: 10.5281/zenodo.4735677 Released 2021-05-01
Analyzed List:	upload_1 (Mus musculus)
Reference List:	Mus musculus (all genes in database)
Test Type:	FISHER
Correction:	FDR

**GO biological process complete**	**Mus musculus - REFLIST (21988)**	**2334**	**expected**	**over/under**	**fold Enrichment**	**raw P-value**	**FDR**
regulation of multicellular organismal process (GO:0051239)	2813	462	298.6	+	1.55	2.90E-19	4.57E-15
cell adhesion (GO:0007155)	848	172	90.01	+	1.91	1.28E-13	1.06E-10

cell differentiation (GO:0030154)	3582	489	380.23	+	1.29	2.43E-08	5.55E-06
regulation of cell differentiation (GO:0045595)	1592	240	168.99	+	1.42	3.67E-07	6.09E-05
positive regulation of cell differentiation (GO:0045597)	938	156	99.57	+	1.57	4.08E-07	6.63E-05
cell morphogenesis involved in differentiation (GO:0000904)	579	102	61.46	+	1.66	6.29E-06	5.90E-04
neuron differentiation (GO:0030182)	1086	167	115.28	+	1.45	1.10E-05	9.69E-04
leukocyte differentiation (GO:0002521)	397	72	42.14	+	1.71	6.70E-05	4.31E-03
cell morphogenesis involved in neuron differentiation (GO:0048667)	449	79	47.66	+	1.66	6.87E-05	4.39E-03
regulation of cell communication (GO:0010646)	3243	499	344.24	+	1.45	4.93E-16	9.73E-13
regulation of localization (GO:0032879)	2870	466	304.65	+	1.53	1.31E-18	1.03E-14
regulation of biological quality (GO:0065008)	3879	589	411.75	+	1.43	3.70E-18	1.94E-14
regulation of response to stimulus (GO:0048583)	3913	589	415.36	+	1.42	2.02E-17	7.97E-14
regulation of signaling (GO:0023051)	3255	501	345.51	+	1.45	4.20E-16	9.45E-13
biological regulation (GO:0065007)	12583	1533	1335.67	+	1.15	2.15E-15	3.76E-12
positive regulation of biological process (GO:0048518)	6225	842	660.78	+	1.27	1.25E-14	1.97E-11
system development (GO:0048731)	4186	602	444.34	+	1.35	3.61E-14	5.18E-11
multicellular organism development (GO:0007275)	4801	674	509.62	+	1.32	4.39E-14	5.77E-11
biological adhesion (GO:0022610)	858	175	91.08	+	1.92	5.08E-14	6.16E-11
regulation of cell migration (GO:0030334)	944	187	100.2	+	1.87	8.03E-14	7.92E-11
response to chemical (GO:0042221)	3492	517	370.67	+	1.39	7.72E-14	8.12E-11
cellular response to chemical stimulus (GO:0070887)	2409	383	255.71	+	1.5	7.65E-14	8.62E-11
regulation of signal transduction (GO:0009966)	2824	434	299.76	+	1.45	1.01E-13	9.33E-11
developmental process (GO:0032502)	5576	761	591.89	+	1.29	1.10E-13	9.63E-11
localization (GO:0051179)	4976	691	528.2	+	1.31	1.57E-13	1.24E-10
positive regulation of multicellular organismal process (GO:0051240)	1595	275	169.31	+	1.62	1.90E-13	1.42E-10

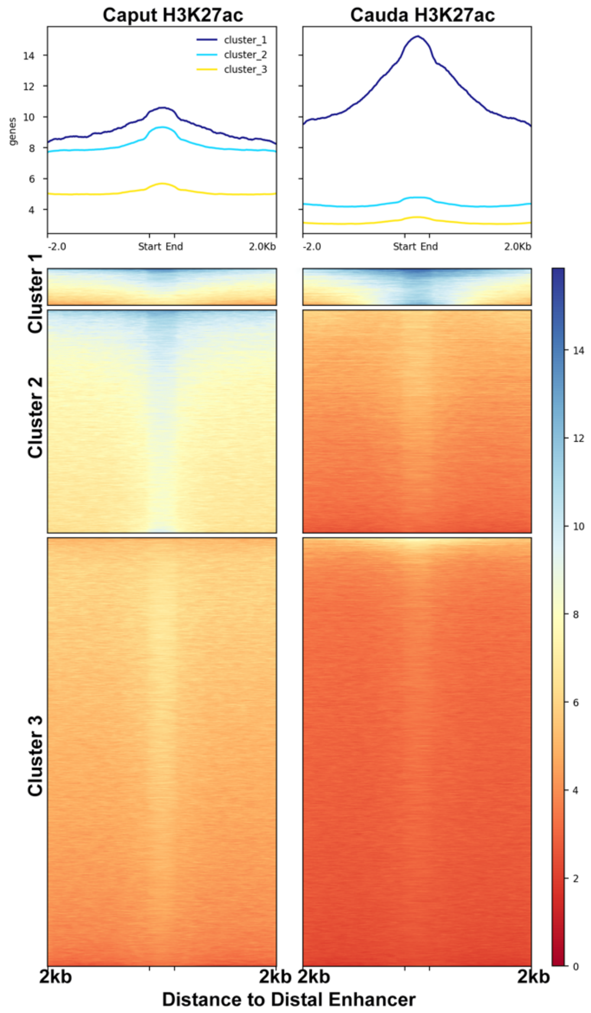

Supplemental Figure 3a Heatmaps displaying the enrichment of H3K27ac signals across regions with a distal enhancer-like signature.

Supplemental Figure 3b. Genomic distribution (top) and top 25 Go Pathways (below) for Cluster 1, CpG Islands exhibiting increased H3K27ac enrichment in cauda-derived sperm.

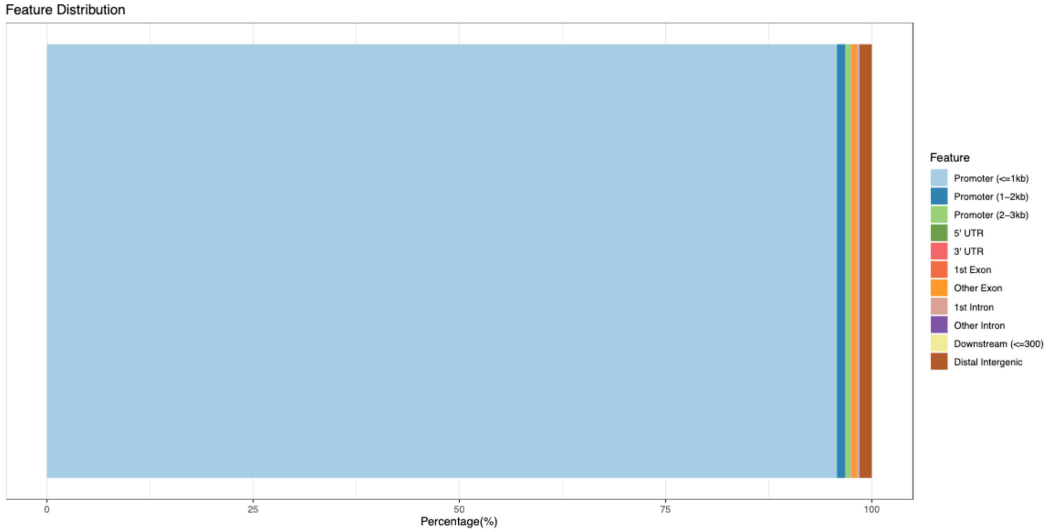

GO biological process complete	Mus musculus - REFLIST (21988)	upload_1 (2439)	upload_1 (expected)	upload_1 (over/under)	upload_1 (fold Enrichment)	upload_1 (raw P-value)	upload_1 (FDR)
histone modification (GO:0016570)	335	94	37.16	+	2.53	2.03E-13	2.46E-11
embryo development (GO:0009790)	1201	202	133.22	+	1.52	7.12E-08	4.75E-06
embryo development ending in birth or egg hatching (GO:0009792)	832	149	92.29	+	1.61	1.60E-07	1.00E-05
regulation of histone modification (GO:0031056)	165	46	18.3	+	2.51	3.47E-07	2.07E-05
sexual reproduction (GO:0019953)	895	148	99.28	+	1.49	1.09E-05	5.11E-04
spermatogenesis (GO:0007283)	586	104	65	+	1.6	1.94E-05	8.61E-04
chromatin remodeling (GO:0006338)	143	35	15.86	+	2.21	8.18E-05	3.15E-03

histone lysine methylation (GO:0034968)	65	21	7.21	+	2.91	8.49E-05	3.26E-03
regulation of histone acetylation (GO:0035065)	58	18	6.43	+	2.8	3.97E-04	1.24E-02
histone H4 acetylation (GO:0043967)	54	17	5.99	+	2.84	4.98E-04	1.49E-02
rRNA processing (GO:0006364)	202	42	22.41	+	1.87	5.13E-04	1.52E-02
reproductive process (GO:0022414)	1480	210	164.17	+	1.28	7.56E-04	2.06E-02
negative regulation of histone modification (GO:0031057)	47	15	5.21	+	2.88	9.31E-04	2.45E-02
embryonic organ morphogenesis (GO:0048562)	326	59	36.16	+	1.63	1.05E-03	2.71E-02
forebrain development (GO:0030900)	385	67	42.71	+	1.57	1.10E-03	2.83E-02
positive regulation of histone methylation (GO:0031062)	45	14	4.99	+	2.8	1.66E-03	4.00E-02
response to stress (GO:0006950)	3231	418	358.4	+	1.17	1.66E-03	4.00E-02
blastocyst development (GO:0001824)	151	32	16.75	+	1.91	1.74E-03	4.14E-02
male gamete generation (GO:0048232)	607	107	67.33	+	1.59	1.99E-05	8.79E-04
cellular metabolic process (GO:0044237)	6410	1139	711.02	+	1.6	1.40E-66	2.21E-62
cellular macromolecule metabolic process (GO:0044260)	3960	810	439.26	+	1.84	3.52E-64	2.77E-60
nitrogen compound metabolic process (GO:0006807)	5748	1027	637.59	+	1.61	2.74E-58	1.44E-54
macromolecule metabolic process (GO:0043170)	5357	973	594.22	+	1.64	3.71E-57	1.46E-53
primary metabolic process (GO:0044238)	6288	1084	697.49	+	1.55	2.00E-55	6.31E-52
cellular protein metabolic process (GO:0044267)	2863	607	317.58	+	1.91	1.23E-49	2.77E-46

Supplemental Figure 4.
Genomic distribution (top) and top 25 Go Pathways (below) for Cluster 1, Enhancer-Like Sequences exhibiting increased H3K27ac enrichment in cauda-derived sperm.

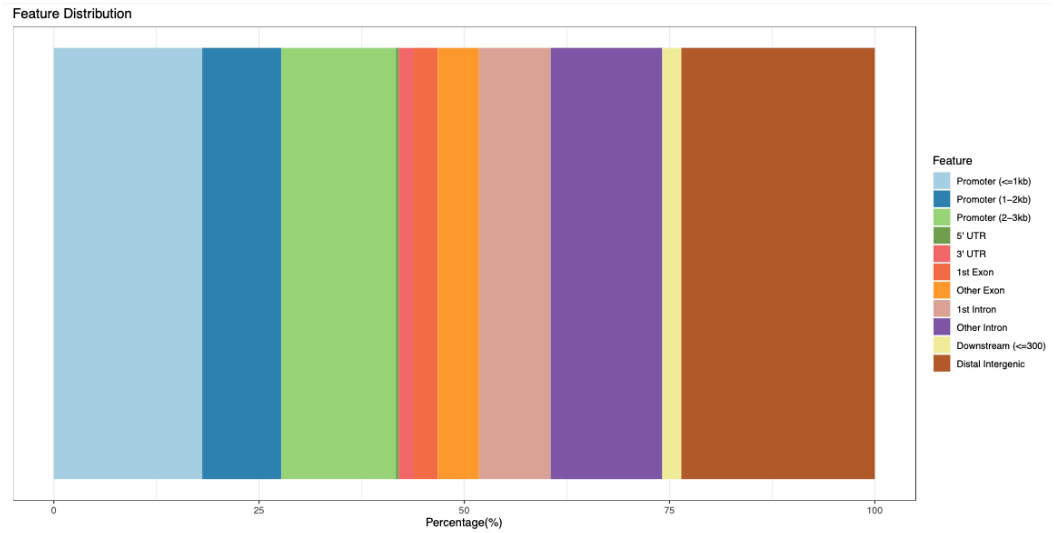

GO biological process complete	Mus musculus - REFLIST (21988)	upload_1 (1697)	expected	over/under	fold Enrichment	raw P-value	FDR
embryo development (GO:0009790)	1201	210	92.69	+	2.27	4.89E-25	1.24E-22
pattern specification process (GO:0007389)	457	101	35.27	+	2.86	3.89E-18	7.14E-16
embryonic morphogenesis (GO:0048598)	627	133	48.39	+	2.75	2.85E-22	6.24E-20
head development (GO:0060322)	705	145	54.41	+	2.66	4.72E-23	1.09E-20
central nervous system development (GO:0007417)	878	172	67.76	+	2.54	4.23E-25	1.09E-22
tube development (GO:0035295)	958	183	73.94	+	2.48	1.19E-25	3.13E-23
embryo development ending in birth or egg hatching (GO:0009792)	832	152	64.21	+	2.37	8.34E-20	1.62E-17
negative regulation of RNA biosynthetic process (GO:1902679)	1249	228	96.4	+	2.37	1.76E-29	5.43E-27

positive regulation of nucleic acid-templated transcription (GO:1903508)	1505	274	116.15	+	2.36	1.31E-35	7.66E-33
positive regulation of transcription, DNA-templated (GO:0045893)	1505	274	116.15	+	2.36	1.31E-35	7.38E-33
positive regulation of RNA biosynthetic process (GO:1902680)	1506	274	116.23	+	2.36	1.41E-35	7.65E-33
lens induction in camera-type eye (GO:0060235)	7	5	0.54	+	9.25	9.70E-04	2.09E-02
negative regulation of apoptotic process involved in morphogenesis (GO:1902338)	7	5	0.54	+	9.25	9.70E-04	2.09E-02
neuron fate determination (GO:0048664)	9	5	0.69	+	7.2	2.17E-03	3.99E-02
forebrain regionalization (GO:0021871)	27	15	2.08	+	7.2	9.81E-08	6.11E-06
cardiac chamber formation (GO:0003207)	12	6	0.93	+	6.48	1.17E-03	2.40E-02
neuron fate commitment (GO:0048663)	74	37	5.71	+	6.48	6.27E-16	1.06E-13
neuron fate specification (GO:0048665)	31	15	2.39	+	6.27	3.87E-07	2.17E-05
embryonic forelimb morphogenesis (GO:0035115)	36	17	2.78	+	6.12	8.60E-08	5.38E-06
neural tube patterning (GO:0021532)	43	16	3.32	+	4.82	2.57E-06	1.22E-04
embryonic cranial skeleton morphogenesis (GO:0048701)	51	16	3.94	+	4.06	1.52E-05	6.19E-04
embryonic eye morphogenesis (GO:0048048)	36	11	2.78	+	3.96	3.88E-04	9.78E-03
embryonic heart tube development (GO:0035050)	87	24	6.71	+	3.57	8.80E-07	4.53E-05
embryonic pattern specification (GO:0009880)	70	17	5.4	+	3.15	1.28E-04	3.96E-03
embryonic digit morphogenesis (GO:0042733)	71	17	5.48	+	3.1	1.48E-04	4.44E-03
post-embryonic development (GO:0009791)	124	28	9.57	+	2.93	3.37E-06	1.56E-04

Supplemental Figure 5.

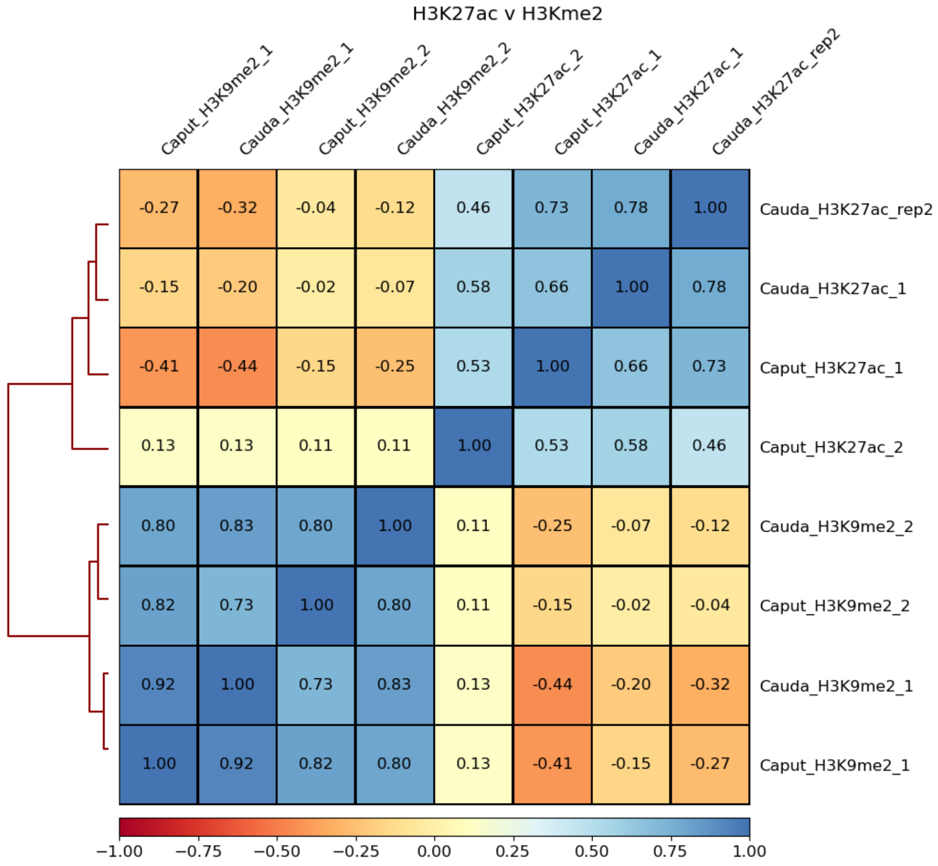

Supplemental Figure 5. Comparison of H3K9me2 and H3K27ac genomic localization reveals mutually exclusive patterns of enrichment. Correlation analysis between H3K9me2 and H3K27ac ChIP-seq signals derived from sperm isolated from the caput and cauda regions of the epididymis (n=2).
